# HIF1α deficiency reduces inflammation in a mouse model of proximal colon cancer

**DOI:** 10.1242/dmm.019000

**Published:** 2015-09-01

**Authors:** Dessislava N. Mladenova, Jane E. Dahlstrom, Phuong N. Tran, Fahad Benthani, Elaine G. Bean, Irvin Ng, Laurent Pangon, Nicola Currey, Maija R. J. Kohonen-Corish

**Affiliations:** 1 Kinghorn Cancer Centre, Garvan Institute of Medical Research, Sydney, New South Wales, 2010, Australia; 2 ACT Pathology, The Canberra Hospital and Australian National University Medical School, Canberra, Australian Capital Territory, 2605, Australia; 3 St Vincent's Clinical School, UNSW Medicine, UNSW Australia, Sydney, New South Wales, 2052, Australia; 4 School of Medicine, University of Western Sydney, Sydney, New South Wales, 2560, Australia

**Keywords:** HIF1α, MIF, AHR, E-cadherin, Sulindac, Colon inflammation

## Abstract

Hypoxia-inducible factor 1α (HIF1α) is a transcription factor that regulates the adaptation of cells to hypoxic microenvironments, for example inside solid tumours. Stabilisation of HIF1α can also occur in normoxic conditions in inflamed tissue or as a result of inactivating mutations in negative regulators of HIF1α. Aberrant overexpression of HIF1α in many different cancers has led to intensive efforts to develop HIF1α-targeted therapies. However, the role of HIF1α is still poorly understood in chronic inflammation that predisposes the colon to carcinogenesis. We have previously reported that the transcription of HIF1α is upregulated and that the protein is stabilised in inflammatory lesions that are caused by the non-steroidal anti-inflammatory drug (NSAID) sulindac in the mouse proximal colon. Here, we exploited this side effect of long-term sulindac administration to analyse the role of HIF1α in colon inflammation using mice with a *Villin-Cre*-induced deletion of *Hif1α* exon 2 in the intestinal epithelium (*Hif1α^ΔIEC^*). We also analysed the effect of sulindac sulfide on the aryl hydrocarbon receptor (AHR) pathway *in vitro* in colon cancer cells. Most sulindac-treated mice developed visible lesions, resembling the appearance of flat adenomas in the human colon, surrounded by macroscopically normal mucosa. *Hif1α^ΔIEC^* mice still developed lesions but they were smaller than in the *Hif1α*-floxed siblings (*Hif1α^F/F^*). Microscopically, *Hif1α^ΔIEC^* mice had significantly less severe colon inflammation than *Hif1α^F/F^* mice. Molecular analysis showed reduced *MIF* expression and increased E-cadherin mRNA expression in the colon of sulindac-treated *Hif1α^ΔIEC^* mice. However, immunohistochemistry analysis revealed a defect of E-cadherin protein expression in sulindac-treated *Hif1α^ΔIEC^* mice. Sulindac sulfide treatment *in vitro* upregulated *Hif1α*, *c-JUN* and *IL8* expression through the AHR pathway. Taken together, HIF1α expression augments inflammation in the proximal colon of sulindac-treated mice, and AHR activation by sulindac might lead to the reduction of E-cadherin protein levels through the mitogen-activated protein kinase (MAPK) pathway.

## Abstract (precis)

**Summary:** HIF1α deficiency reduces inflammation in the mouse proximal colon but is associated with defective E-cadherin expression in colon epithelial cells when mice lacking intestinal epithelium expression of *Hif1α* are challenged with sulindac.

## INTRODUCTION

The gastrointestinal tract adapts to rapid and drastic changes in tissue oxygen availability (Colgan and Taylor, 2010). Hypoxia-inducible factor (HIF) plays a key role in promoting cell survival under hypoxic conditions. HIF also has a central protective function in maintaining the gut epithelial barrier (Colgan and Taylor, 2010). HIF is rapidly degraded upon oxygen availability and is stabilised in hypoxic conditions. However, HIF is also an inflammatory mediator that can be stabilised under normoxia, as well as other conditions such as the upregulation of pro-inflammatory IL-1β (Jung et al., 2003).

HIF is a heterodimeric transcription factor and comprises a constitutively expressed HIF1β (also known as aryl hydrocarbon receptor nuclear translocator, ARNT) subunit and one of several inducible HIFα subunits, the best characterised of which are HIF1α and the closely related HIF2α (Semenza, 2003). The role of HIF in colon inflammation is still poorly understood. HIF1α expression in the colon mucosa is protective in the trinitrobenzene sulfonic acid (TNBS) and oxazalone models of murine colitis. In these models, the protective function of HIF1α is mediated through upregulation of barrier protective genes and increased resistance to injury in the colon mucosa (Karhausen et al., 2004). HIF1α that is expressed by T cells is also protective in the dextran sodium sulfate (DSS) model of colitis (Higashiyama et al., 2012). By contrast, colon-specific constitutive expression of HIF (through Vhl disruption) augments colitis and increases cytokine expression in the DSS model (Shah et al., 2008).

Mouse models have been used to identify many factors that can initiate or contribute to chronic inflammation in the colon, such as disruption of intercellular signalling, and cross-talk between epithelial and inflammatory cells or injury to the mucosal barrier (Mladenova and Kohonen-Corish, 2012). We have previously shown that HIF1α transcription is upregulated and the protein level is stabilised in inflammatory lesions of the mouse colon, which are caused by the non-steroidal anti-inflammatory drug (NSAID) sulindac (Mladenova et al., 2011). Sulindac is a drug used to treat arthritis that can also be prescribed as a cancer chemoprevention agent, but its use is limited by gastrointestinal side effects. In the mouse, long-term administration of oral sulindac prevents carcinogen-induced cancer in the distal colon but also causes small foci of mucosal surface damage to the proximal colon. These can progress to visible lesions that exhibit acute and chronic inflammation (Mladenova et al., 2011). Microscopic foci of mucosal damage are evident after 1 week of sulindac diet. After 20 weeks of exposure to sulindac, the macroscopic lesions resemble flat adenomas in the human colon. The presence of early microscopic surface erosions suggests that damage to the mucosal barrier plays a role in this model. In knockout mice with defective tumour suppressor genes *Msh2*, *p53* (Mladenova et al., 2011), *Mlh1* or *Apc* (Itano et al., 2009), the inflammatory damage can further progress to cancer. However, the number and size of the visible lesions, as well as the severity of inflammation is comparable between the knockout mice and their wild-type siblings, despite the difference in neoplasia frequency (Mladenova et al., 2011).

**TRANSLATIONAL IMPACT**

**Clinical issue**

Aberrant overexpression of hypoxia-inducible factor 1α (HIF1α) in many different cancers has led to intensive efforts to develop HIF1α-targeted therapies. However, HIF1α is also important in protecting the colon mucosal barrier, and the role of HIF1α is poorly understood in chronic inflammation that predisposes the colon to carcinogenesis. Previous studies have shown that administration of the chemoprevention agent sulindac in mice prevents carcinogen-induced cancer in the distal colon but also causes early foci of mucosal damage to the proximal colon, which can progress to visible lesions with acute and chronic inflammation. The presence of surface erosion suggests that damage to the colon mucosal barrier plays a role in sulindac-induced inflammation. Transcription of Hif1α is upregulated, and the protein is stabilized in sulindac-induced inflammatory lesions. Here, the authors have exploited the side effect of long-term sulindac administration on the proximal colon to analyse the role of HIF1α in colon inflammation using mice that lack *Hif1α* in the intestinal epithelium (*Hif1α^ΔIEC^*).

**Results**

Sulindac-treated *Hif1α^ΔIEC^* mice developed colonic lesions but these were smaller than those detected in the genotype control mice (*Hif1α^F/F^*) treated with the same drug. Microscopically, *Hif1α^ΔIEC^* mice had significantly less severe colon inflammation than *Hif1α^F/F^* mice. Loss of HIF1α reduced the expression of the macrophage migration inhibitor factor (MIF) in sulindac-treated mice, which is compatible with the observed reduction in the severity of inflammation. HIF is a negative regulator of the cell-adhesion protein E-cadherin (reduced expression of which is associated with invasiveness in human carcinomas) and as expected, loss of HIF1α increased E-cadherin mRNA expression in the colon. E-cadherin protein expression was also increased in *Hif1α^ΔIEC^* mice, but treatment with sulindac abolished such an increase.

**Implications and future directions**

These results indicate that HIF1α expression augments inflammation in the proximal colon of sulindac-treated mice. By contrast, HIF1α expression might be protective against sulindac-induced reduction in E-cadherin protein expression. These findings provide *in vivo* evidence of the dual role of HIF1α in the colon. As sulindac is a known activator of the aryl hydrocarbon receptor signalling pathway that regulates detoxification of many environmental contaminants and drugs, further studies of this model might be informative in understanding the diverse tissue-specific effects of this important drug detoxification pathway.

We have exploited this exaggerated inflammatory response to sulindac in the mouse proximal colon in order to analyse the role of HIF1α in colon inflammation. Here, we generated mice with specific knockout of HIF1α in the colon and small intestine epithelium (*Hif1α^ΔIEC^*) using the *Cre* recombinase transgene under the control of the *Villin* promoter (Madison et al., 2002). We also further analysed the effects of treatment with sulindac sulfide on the expression of pro-inflammatory genes, using our previously established *in vitro* model (Mladenova et al., 2011, 2013).

## RESULTS

### Colon-specific deficiency of *Hif1α* is protective against sulindac-diet-induced mucosal inflammation

We have previously described that administration of a long-term sulindac diet causes similar levels of tissue damage to the colon in tumour suppressor gene knockout mice compared with their wild-type siblings (Mladenova et al., 2011). Sulindac-induced lesions are localised to a specific region of the proximal colon (labelled thereafter as P2), and show high levels of inflammation and high expression of HIF1α. Therefore, we generated mice with a colon-specific deletion of HIF1α (*Hif1α^ΔIEC^*) using the *Villin-Cre* mouse line (Madison et al., 2002). The efficiency of Cre-mediated recombination of the HIF1α-lox allele was 91% in the proximal colon mucosa of *Hif1α^ΔIEC^* mice.

**Fig. 1.**
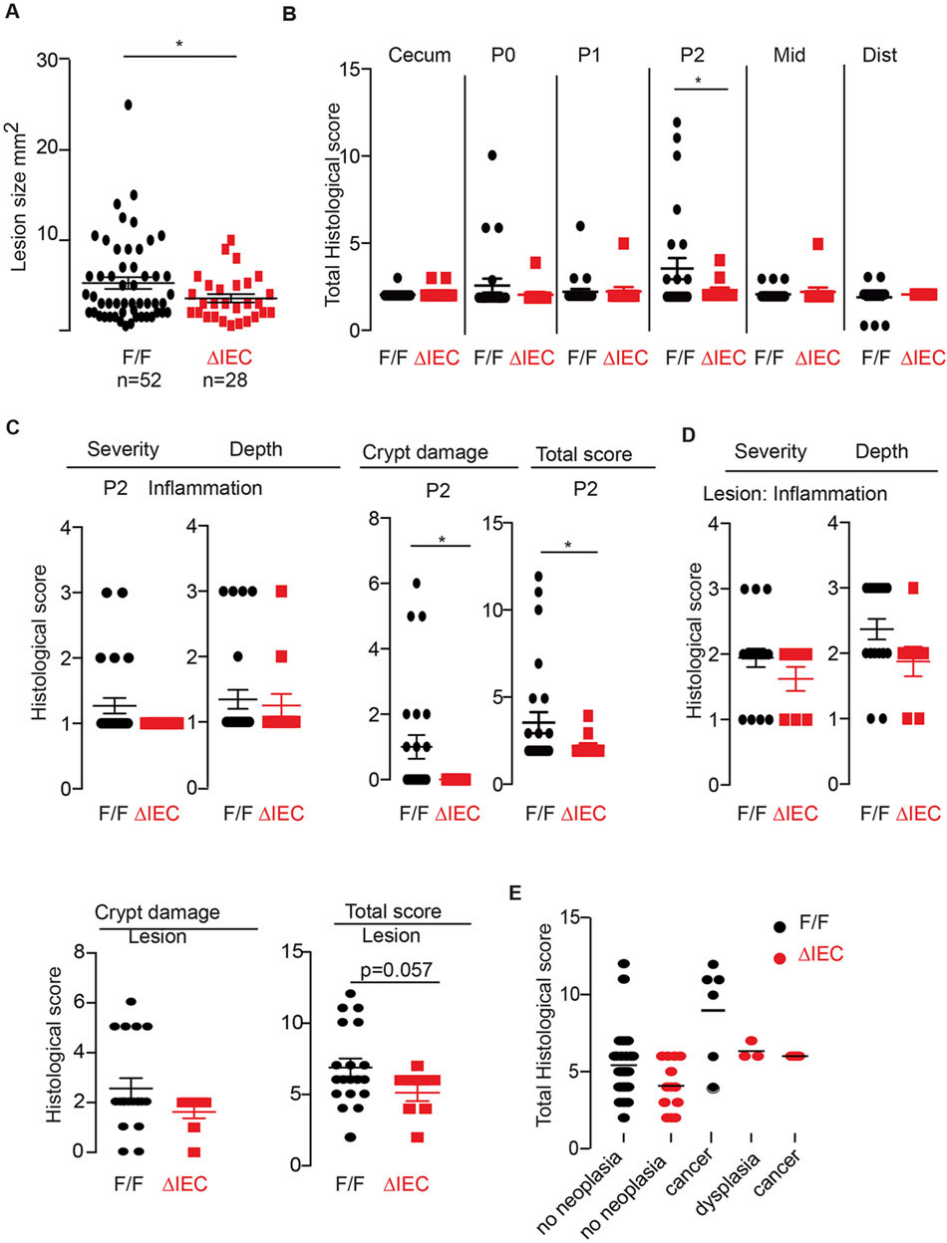
**Sulindac-treated *Hif1α^ΔIEC^* mice develop smaller lesions and less mucosal inflammation in the colon compared with control *Hif1α^F/F^* mice.** (A) Average size of individual lesions±s.e.m. (B) Total inflammation score in different regions of the colon (outside lesions). *Hif1α^F/F^ n*=26, *Hif1α^ΔIEC^ n*=12. (C) Severity and depth of inflammation and crypt damage in the proximal P2 region (outside lesions) of the colon. *Hif1α^F/F^ n*=26, *Hif1α^ΔIEC^ n*=12. (D) Severity and depth of inflammation, crypt damage and the total inflammation score in the pathologically most severe lesion for each mouse. *Hif1α^F/F^ n*=19, *Hif1α^ΔIEC^ n*=8. (E) The total inflammation score of all dissected lesions from *Hif1α^ΔIEC^* and *Hif1α^F/F^* mice plotted against the pathology assessment for neoplasia (dysplasia or cancer). *Hif1α^ΔIEC^* and *Hif1α^F/F^* mice were given 320 ppm sulindac in the diet for 20 weeks, after which biopsies were taken from the flat mucosa and lesions along the entire length of the colon and assessed by a pathologist as described in Materials and Methods. The bars represent the average histological score±s.e.m. The total histological score is a sum of the three individual scores for severity of inflammation, depth of inflammation and crypt damage. Star (*) indicates *P*<0.05. ΔIEC, *Hif1α^ΔIEC^*; F/F, *Hif1α^F/F^*; Mid, middle colon; Dist, distal colon.

We first determined whether *Hif1α* deficiency in the colon affected the severity of sulindac-induced mucosal inflammation. After 20 weeks of sulindac treatment, the colons were harvested and analysed at the macroscopic level. Visible inflammatory lesions were carefully measured with a fitted eyepiece grid, and their size and location in the colon recorded. *Hif1α^ΔIEC^* mice still developed visible lesions in the P2 region of the proximal colon (Mladenova et al., 2011), but the individual lesions were significantly smaller in *Hif1α^ΔIEC^* mice compared with those in *Hif1α-*floxed mice (*Hif1α^F/F^*) mice (*P*=0.037) (Fig. 1A). The *Hif1α^ΔIEC^* mice also developed fewer large lesions (>10 mm^2^) (3.6% vs 17%, *P*=not significant). Three out of 26 *Hif1α^F/F^* mice (12%) had macroscopic colon inflammation outside the lesions, involving large areas of the colon, with the affected area in these three cases estimated to cover 10, 15 and 5% of the whole colon, respectively. No *Hif1α^ΔIEC^* mice showed macroscopic damage outside of the lesions.

**Fig. 2.**
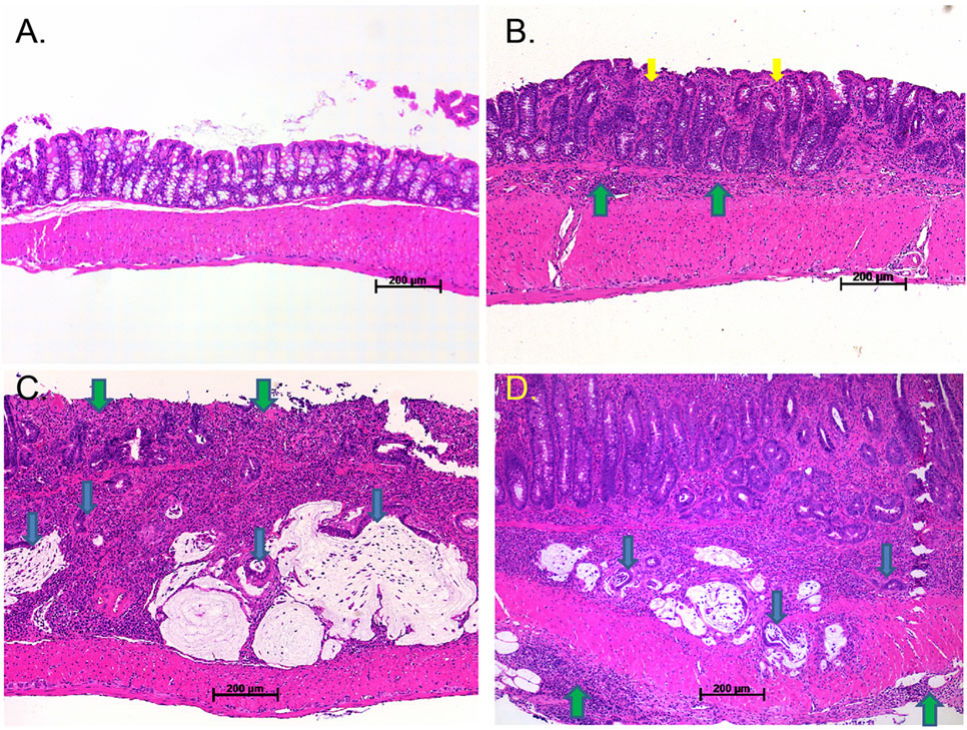
**Photomicrographs of sulindac-diet-induced colon inflammation and cancer.** (A) Hematoxylin and eosin (H&E)-stained sections of proximal colon from the P2 region of a control-fed *Hif1α^F/F^* mouse that appeared to be macroscopically normal. (B) Mild-active moderate chronic inflammation in a proximal lesion harvested from a sulindac-treated *Hif1α^ΔIEC^* mouse. The inflammation is confined to the mucosa and submucosa. Yellow arrows indicate mucosal inflammation. Green arrows indicate submucosal inflammation. (C) Well-differentiated mucinous adenocarcinoma arising in an area of moderate-active moderate chronic inflammation with mucosal erosion in a *Hif1α^ΔIEC^* mouse. Blue arrows indicate islands of adenocarcinoma. Green arrows indicate mucosal erosion. (D) Well-differentiated adenocarcinoma extending into the muscularis propria arising in an area of moderate-active severe chronic inflammation in a *Hif1α^F/F^* mouse. Note the inflammation extends into the adventia. Blue arrows indicate islands of adenocarcinoma. Green arrows indicate adventitial inflammation.

We then analysed the mucosal surface microscopically by scoring the severity of inflammation, depth of inflammation and crypt damage, which were combined to produce a total inflammation score. *Hif1α^ΔIEC^* mice had significantly less-severe colon inflammation in the mucosa between lesions in the P2 region compared with mice from the control genotype (Fig. 1B,C), including the score for crypt damage and the total inflammation score (*P*<0.05). Based on the histological score, the most severely inflamed lesion for each mouse was chosen for further analysis, and there was a trend for lower total inflammation scores in the *Hif1α^ΔIEC^* mice compared with *Hif1α^F/F^* mice (*P*=0.057; Fig. 1D). As in our previous experiment, a subset of sulindac-induced lesions in the colon progressed to adenocarcinoma. The inflammatory microenvironment is rich in mutagenic reactive oxygen species and pro-inflammatory factors that can promote cancer initiation and growth, and lead to tissue remodelling (Mladenova and Kohonen-Corish, 2012). Surprisingly, the frequency of colon adenocarcinoma was similar in *Hif1α^ΔIEC^* (18%; two out of 11) and *Hif1α^F/F^* mice (16%; four out of 25) although there was less mucosal inflammation in the *Hif1α^ΔIEC^* mice. The distribution of total inflammation scores in neoplastic and non-neoplastic lesions is shown in Fig. 1E. The photomicrographs in Fig. 2 and supplementary material Figs S1 and S2 show some of the typical pathological changes observed in the proximal colon of sulindac-treated mice, ranging from mild inflammation with no neoplasia to moderate and severe inflammation with dysplasia and adenocarcinoma. A subset of sulindac-fed mice also developed small foci of acute or chronic hepatitis. Sulindac is a rare but known cause of hepatitis in humans (Wood et al., 1985).

### *Hif1α-*knockout defect causes downregulation of macrophage migration inhibitor factor, MIF, but increases *Hif2α* mRNA levels in sulindac-treated colon mucosa

**Fig. 3.**
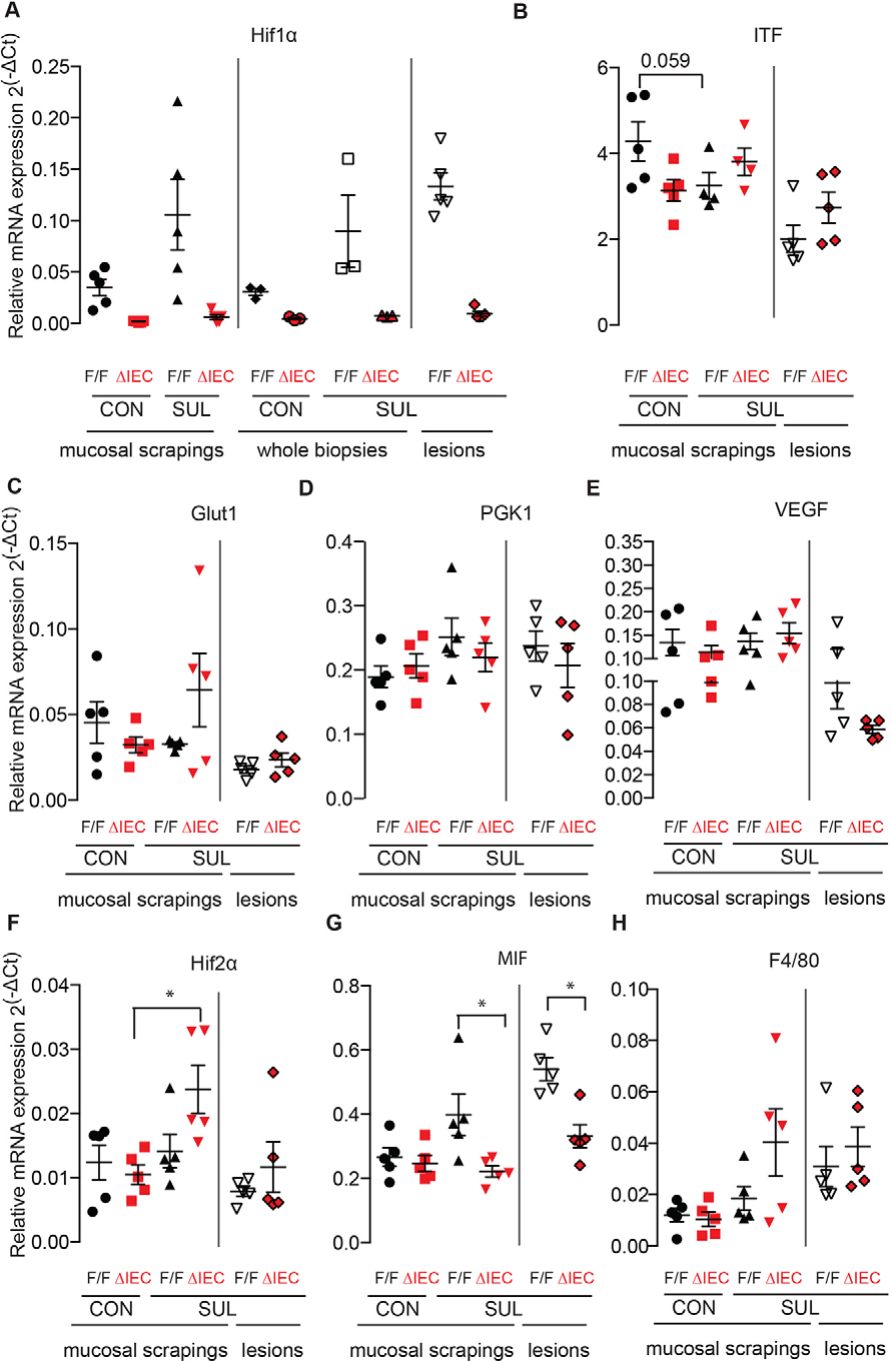
**Treatment with sulindac upregulates HIF2α and downregulates MIF in *Hif1α^ΔIEC^* mice compared with *Hif1α^F/F^* mice.** Q-PCR analysis for gene expression of *Hif1α* (A), *ITF* (B), *Glut1* (C), *Pgk1* (D), *Vegfa* (E), *Hif2α* (F), *MIF* (G) and the macrophage marker F4/80 (H). RNA was extracted from the mucosal scrapings, and lesions were harvested from the proximal colon of *Hif1α^ΔIEC^* and *Hif1α^F/F^* mice that had been treated with sulindac (Sul) or the control (Con) diet. The q-PCR primers for *Hif1α* span exons 2 and 3. Gene expression was normalised to that of *Rpl19*. Error bars indicate s.e.m. Star (*) indicates *P*<0.05.

We observed a 19-fold reduction of *Hif1α* mRNA expression in *Hif1α^ΔIEC^* mice compared with that of the control genotype (Fig. 3A). The generation of *Hif1α^ΔIEC^* involves deletion of exon 2, which contains sequences encoding the basic helix-loop-helix (bHLH) domain that is essential for DNA binding and dimerisation of HIF1α and HIF1β (Jiang et al., 1996), and that results in downregulation of HIF1α-target genes (Iyer et al., 1998; Ryan et al., 1998). As we have previously shown in wild-type mice, the sulindac diet increased HIF1α expression in *Hif1α^F/F^* mice, and this effect was most pronounced in the inflammatory colon lesions (Fig. 3A). HIF1α expression remained low in the colon of the *Hif1α^ΔIEC^* mice that had been treated with sulindac.

We next examined the HIF1α-target genes phosphoglycerate kinase-1 (*Pgk1*), glucose transporter (*Glut1*), vascular endothelial growth factor (*Vegfa*) and intestinal trefoil factor (*ITF*) (Fig. 3B-E) (Iyer et al., 1998; Karhausen et al., 2004; Ryan et al., 1998). None of the examined genes were significantly downregulated in the *Hif1α^ΔIEC^* when compared with their levels in *Hif1α^F/F^* mice. *Glut1* and *Vegfa* genes are under the transcriptional control of both HIF1α and HIF2α (Raval et al., 2005). The HIF2α subunit of hypoxia-inducible factor can also drive hypoxia-dependent responses, and HIF1α and HIF2α have been shown to regulate common target genes (Raval et al., 2005). We next assessed the level of *Hif2α* by using quantitative PCR (q-PCR). The sulindac diet increased *Hif2α* expression levels in the colon mucosa of *Hif1α^ΔIEC^* but not of *Hif1α^F/F^* mice or in the lesions (Fig. 3F). Therefore, the lack of downregulation of HIF1α-target genes in the colon of *Hif1α^ΔIEC^* mice might be due to the compensatory role of HIF2α and/or other transcription factors in regulating these genes.

We have previously shown that pro-inflammatory genes such as *IL-1β*, *MIP-2* and *Cox-2* are upregulated by the sulindac diet in the mouse proximal colon (Mladenova et al., 2011, 2013), and impairment in their induction could explain the reduction in the inflammatory response. However, the level of induction of these cytokines was not significantly different between *Hif1α^ΔIEC^* and *Hif1α^F/F^* mice (data not shown). We next examined whether the lower levels of inflammation observed in the colon of *Hif1α^ΔIEC^* mice were associated with changes in macrophage infiltration or expression of the macrophage migration inhibitory factor (*MIF*). HIF1α is essential for the function, migration, motility and invasiveness of myeloid cells (Cramer et al., 2003; Scortegagna et al., 2008), and *MIF* is a direct target of HIF1. MIF is a crucial mediator of HIF-induced pro-inflammatory responses in a mouse model of experimentally induced colitis (Baugh et al., 2006; Cramer et al., 2003; Scortegagna et al., 2008; Shah et al., 2008). Sulindac-treated *Hif1α^ΔIEC^* mice showed significantly lower expression of *MIF* expression in the colon compared with *Hif1α^F/F^* mice (Fig. 3G). These results are consistent with the previously reported MIF-dependent, pro-inflammatory role of HIF, although in that study the effect was primarily attributed to HIF2α (Shah et al., 2008). Expression of the macrophage-specific marker F4/80 tended to increase with the sulindac diet, but there were no significant differences between the two genotypes (Fig. 3H).

### Sulindac-treated *Hif1α^ΔIEC^* mice show a defect in E-cadherin protein expression in the colon mucosa

Activation of HIF1α can lead to transcriptional repression of the key cell adhesion protein E-cadherin (Esteban et al., 2006). Reduced membrane expression of E-cadherin is a hallmark of the epithelial-mesenchymal transition (EMT) (Lamouille et al., 2014). Recently, it has been shown that in a mouse model of colorectal cancer driven by *Apc* allelic loss, early and late neoplasms exhibit defects in epithelial barrier maintenance, leading to microbial invasion, which triggers tumour-elicited inflammation and contributes to tumour growth (Grivennikov et al., 2012). Polymorphisms in E-cadherin, which is encoded by the *CDH1* gene, are also associated with the pathogenesis of inflammatory bowel disease (IBD), suggesting that defects in the epithelial barrier and adherent junctions might contribute to the development of IBD (Muise et al., 2009). Despite the reduced level of inflammation, a small percentage of the *Hif1α^ΔIE^* mice still developed neoplasia, similar to *Hif1α^F/F^* mice, after receiving the sulindac diet. Therefore, we next examined whether the *Hif1α^ΔIEC^* defect caused changes in E-cadherin gene expression.

**Fig. 4.**
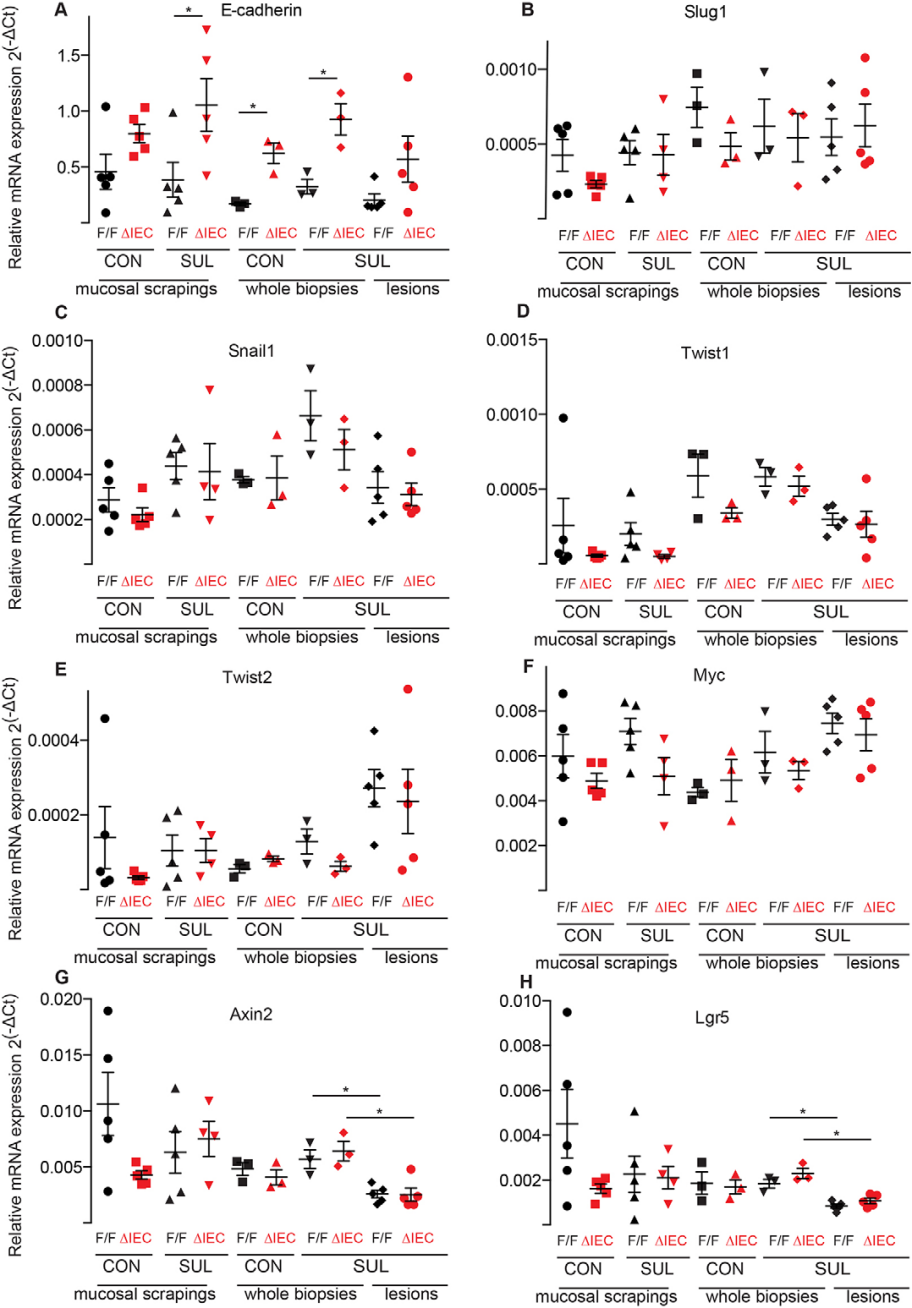
**HIFα deficiency increases E-cadherin mRNA expression in the colon mucosa of *Hif1α^ΔIEC^* mice.** Q-PCR analysis for mRNA expression of E-cadherin (A), *Slug* (*Snai2*) (B), *Snail* (*Snai1*) (C), *Twist1* (D), *Twist2* (E), *Myc* (F), *Axin2* (G) and *Lgr5* (H) in the mucosal scrapings, whole biopsies and lesions harvested from the proximal colon of *Hif1α^ΔIEC^* and *Hif1α^F/F^* mice treated with sulindac (Sul) or the control (Con) diet. Gene expression was normalised to that of *Rpl19*. Error bars indicate s.e.m. Star (*) indicates *P*<0.05. ΔIEC=*Hif1α^ΔIEC^*, F/F=*Hif1α^F/F^*.

Q-PCR analyses showed that mRNA levels of E-cadherin were significantly higher in *Hif1α^ΔIEC^* mice compared with those of *Hif1α^F/F^* mice (Fig. 4A). The increase was seen in mice that received either the control or the sulindac diet, and therefore, it was due to the loss of HIF1α in the colon epithelium. E-cadherin gene expression is repressed through EMT transcription factors, such as Snail, Slug and Twist (Batlle et al., 2000; Bolos et al., 2003; Cano et al., 2000; Hajra et al., 2002; Yang et al., 2008). However, no significant differences were observed in the expression of *Snail*, *Slug* or *Twist* between *Hif1α^F/F^* and *Hif1α^ΔIEC^* mice (Fig. 4B-E).

**Fig. 5.**
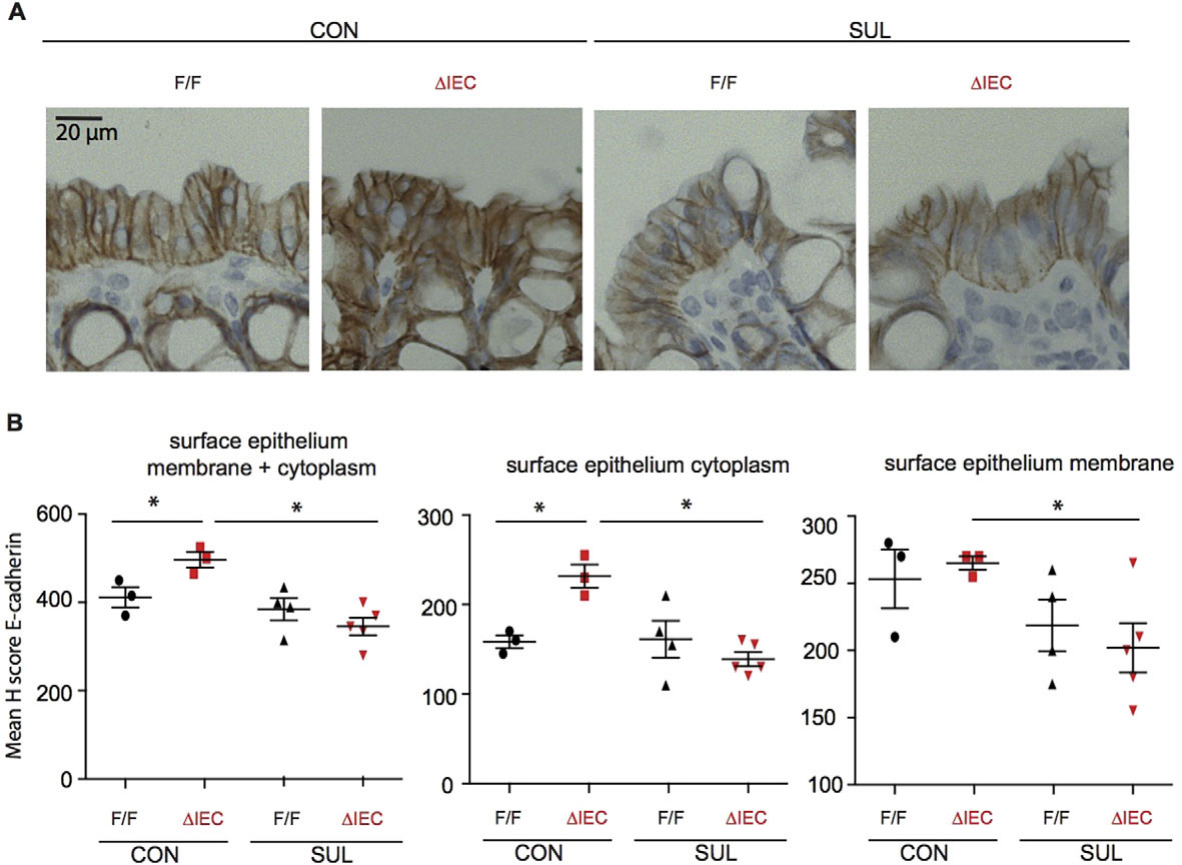
**HIF1α deficiency predisposes the colon mucosa to a defect in E-cadherin protein expression in sulindac-treated mice.** (A) Representative photomicrographs of E-cadherin staining in the P2 region of *Hif1α^ΔIEC^* and *Hif1α^F/F^* mice treated with sulindac (Sul) or the control (Con) diet. (B) Quantification of the staining intensity of E-cadherin in the cytoplasm and membrane of colon epithelial cells using the H-score. Error bars indicate s.e.m. Star (*) indicates *P*<0.05. ΔIEC=*Hif1α^ΔIEC^*; F/F=*Hif1α^F/F^*.

E-cadherin is detected in both the cytoplasm and the membrane of colon epithelial cells. Membrane-bound E-cadherin is crucial in maintaining the integrity of the intercellular junctions. We next determined whether sulindac changed E-cadherin protein expression in the mucosa of the proximal colon, which is most affected by sulindac-induced epithelial damage (Fig. 5). Consistent with the results of mRNA analyses, E-cadherin protein levels were higher in *Hif1α^ΔIEC^* mice compared with those in *Hif1α^F/F^* mice, but this was only seen in the mice that received the control feed. Remarkably, with sulindac exposure, there was a clear decrease in E-cadherin protein level in *Hif1α^ΔIEC^* mice despite an increase in E-cadherin mRNA level (Fig. 4A). This was due to reduced expression of both cytoplasmic and membrane-bound E-cadherin. Thus, although the *Hif1α^ΔIEC^* mice showed less inflammatory damage in the colon following treatment with sulindac, these mice displayed a defect in E-cadherin protein expression. This suggests that E-cadherin protein expression in the proximal colon is reduced by sulindac and that this is more pronounced in the absence of HIF1α.

Sulindac metabolites are known to cause degradation of β-catenin in colon cancer cells (Rice et al., 2003). Therefore, we next analysed the expression of the RNA of β-catenin-target genes. There was no difference between *Myc*, *Axin2* or *Lgr5* expression between *Hif1α^ΔIEC^* and *Hif1α^F/F^* mice (Fig. 4F-H). However, there was reduced expression of *Axin2* and *Lgr5* in the inflammatory lesions that had been induced by sulindac, regardless of genotype.

### Treatment of colon cancer cells with sulindac sulfide *in vitro* causes upregulation of inflammation and cancer-promoting genes through AHR.

**Fig. 6.**
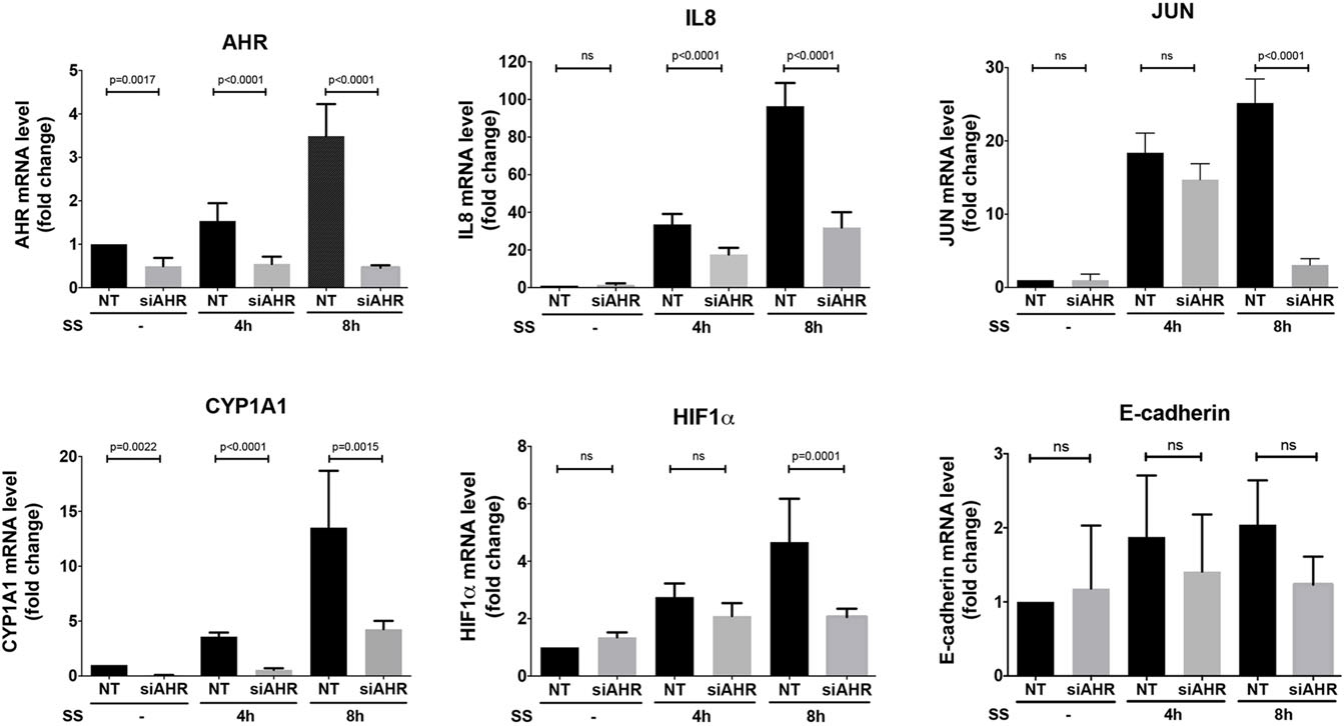
**Sulindac sulfide upregulates *Hif1α*, *CYP1A1*, *c-JUN* and *IL8* through activation of AHR.** Q-PCR analysis for mRNA expression of *AHR*, *IL8*, *c-JUN*, *CYP1A1*, *Hif1α* and E-cadherin * *in HCT15 cells that had been treated with 50 μM sulindac sulfide (SS) for 4 h or 8 h. Gene expression was normalised to that of *GAPDH*. Error bars indicate s.e.m. *P*-values were calculated using Student's *t*-test. ns, not significant.

**Fig. 7.**
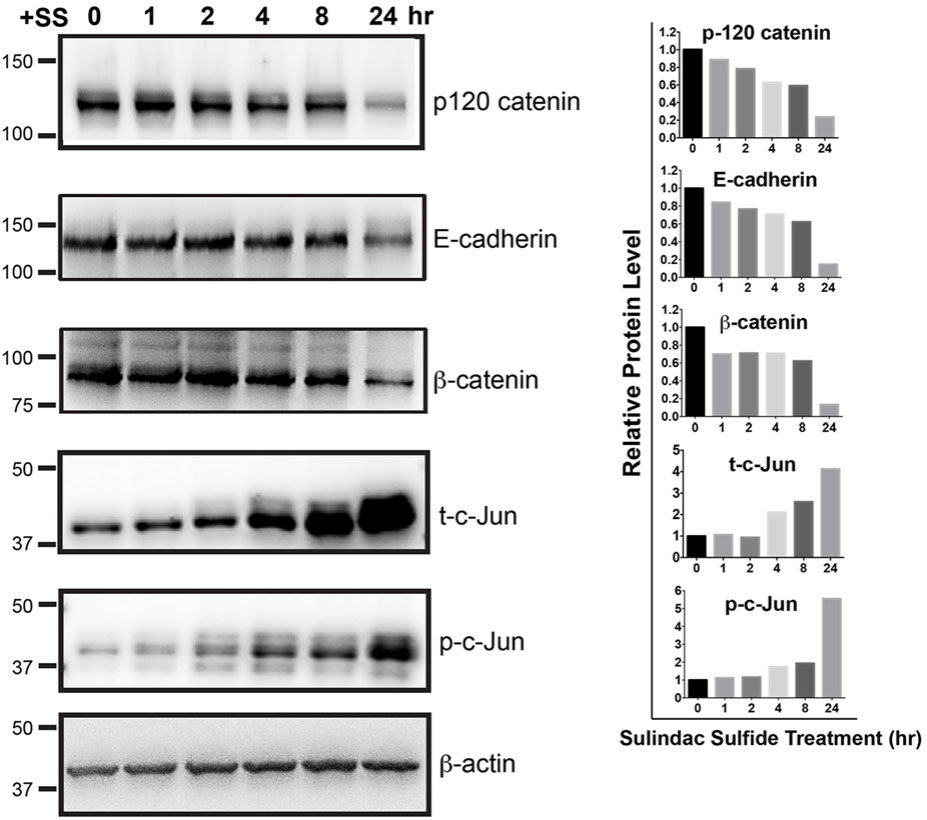
**Treatment of HCT15 cells with sulindac sulfide causes an increase in the level of total and phosphorylated c-Jun and a decrease of E-cadherin, β-catenin and p120-catenin levels.** Western blot was performed on protein extracts from HCT15 cells treated with 50 μM sulindac sulfide (SS) over a time-course. Specific antibodies were used to detect the protein level of p120-catenin, E-cadherin, β-catenin, total c-Jun (t-c-Jun) and phosphorylated c-Jun (p-c-Jun). The membranes were also probed with an antibody against β-actin as a loading control. The relative intensity of protein bands was quantified using ImageJ 1.48v software (National Institutes of Health). The protein expression levels were normalised to the loading control β-actin and relative to the no-sulindac-sulfide treatment (0 hr).

We have previously shown that the sulfide metabolite of sulindac can induce NF-κB and AP-1 (c-Jun and JunD) signalling in colon cancer cells, which lead to upregulation of the chemokine IL8 (Mladenova et al., 2011, 2013). This resembles the activation of the aryl hydrocarbon receptor (AHR) signalling pathway that regulates detoxification of many environmental contaminants and pharmacological drugs but that can also lead to activation of inflammatory cytokines (Fardel, 2013; Safe et al., 2013). Sulindac is a known ligand and activator of AHR (Ciolino et al., 2006). Therefore, we next tested whether sulindac can activate AHR-associated pathways in our *in vitro* model. HCT15 cells were treated with 50 µM sulindac sulfide for 1-24 h. Q-PCR analysis of gene expression showed that sulindac sulfide upregulated *CYP1A1*, the prototype phase I response target of AHR, as well as *c-JUN*, *IL8* and *Hif1α*. The upregulation of all four genes was abolished with AHR knockdown (Fig. 6). This suggests that sulindac can activate AHR, leading to activation of c-Jun signalling, which is a known EMT-promoting transcription factor. AHR activation through dioxin, the well-known AHR ligand, has been previously shown to lead to loss of cell-cell adhesion in MCF7 breast epithelial cells (Diry et al., 2006). In our model, sulindac-sulfide-induced phosphorylation of c-Jun increased until 24 h, whereas there was a gradual decrease in E-cadherin, β-catenin and p120-catenin levels (Fig. 7).

## DISCUSSION

The role of HIF in promoting carcinogenesis has been extensively studied, but its role in intestinal inflammation is poorly understood (Bracken et al., 2003; Semenza, 2003). Amongst the factors contributing to inflammatory bowel disorders are dysregulation and inappropriate activation of inflammatory signalling, and defects in the mucosal barrier. HIF1 signalling has been reported to play a role in both the immune response and in the maintenance of the integrity of the mucosal epithelial barrier (Mladenova and Kohonen-Corish, 2012). Epithelial cells are now seen as important players in the gut immune response because they have the ability to secrete a range of pro-inflammatory factors and other mediators that modulate the function of the immune cells (Mladenova and Kohonen-Corish, 2012). Constitutive expression of HIF1α in basal epidermal keratinocytes in transgenic mice primes an increase in inflammatory infiltrate (Scortegagna et al., 2008). Similarly, HIF overexpression in colon epithelial cells exacerbates DSS-induced colitis, and results in an increased inflammatory infiltrate and colon oedema, even without treatment with DSS (Shah et al., 2008). Consistent with these data, in our previous study, we showed that treatment with sulindac induced significantly less colon inflammation in *Hif1α^Δ/Δ^*^*IEC*^ mice (Mladenova et al., 2011). Those mice had a *Villin*-*Cre* driven deletion of *Hif1α* in the intestine and a heterozygous *Hif1α*-null mutation in the whole body – i.e. including the inflammatory cells infiltrating the colon mucosa.

Here, we deleted HIF1α specifically in mouse intestinal epithelium and achieved a good efficiency of recombination (91%). *Hif1α* mRNA levels were significantly reduced in *Hif1α^ΔIEC^* mice compared with those in mice that expressed HIF1α from the floxed allele (*Hif1α^F/F^*). However, *Hif2α* mRNA levels were not affected in *Hif1α^ΔIEC^* mice, and *Hif2α* levels were significantly increased in the colons of *Hif1α^ΔIEC^* mice that were fed with the sulindac diet when compared with those of mice on the control diet. HIF1α and HIF2α regulate overlapping or distinct sets of target genes in different tissues (Raval et al., 2005). Therefore, it is possible that HIF2α expression compensates to some extent for the lack of HIF1α expression in the colon mucosa. In support of this hypothesis, we did not observe downregulation of the HIF-target genes *Glut1*, *Pgk1*, *Vegfa*, *ITF*, *Snail* or *Twist*.

As we have previously reported, treatment of mice with sulindac triggers colon inflammation and the formation of visible lesions in the proximal colon, most pronounced in a region that we designated as P2 (Mladenova et al., 2011). Histopathology analysis showed that deficiency of HIF1α in the colon epithelium resulted in a decrease in the inflammatory response in both the macroscopically normal (uninvolved) proximal P2 mucosa and in the lesions. The reduction of the inflammatory response was accompanied by a significant decrease in *MIF* expression in *Hif1α^ΔIEC^* mice. *MIF* is a direct target of HIF1α (Baugh et al., 2006) and is described as a link between inflammation and cancer, contributing to a microenvironment favouring cancer progression (Conroy et al., 2010). MIF has been found to play a role in a mouse model of experimentally induced colitis, and MIF plasma concentrations are increased in individuals with Crohn's disease (de Jong et al., 2001). Similarly, transgenic mice that overexpress MIF are more susceptible to DSS-induced colitis (Ohkawara et al., 2005). Conversely, antibodies against MIF ameliorate DSS-induced colitis in mice (Ohkawara et al., 2002). Shah and colleagues have reported that HIF expression exacerbates colitis through a MIF-dependent mechanism (Shah et al., 2008). Therefore, we speculate that reduced MIF expression contributes to the reduction of colon inflammation in *Hif1α^ΔIEC^* mice.

HIF is one of the known factors promoting EMT in tumour cells and is a negative regulator of E-cadherin expression, potentially through several mechanisms (Esteban et al., 2006; Lamouille et al., 2014). Membrane-bound E-cadherin plays an important role in maintaining the homeostasis of the gut epithelial lining to prevent invasion of pathogenic bacteria (Schneider et al., 2010). Reduced membrane E-cadherin is also observed in the development of invasive carcinomas. As expected, gut-specific ablation of *Hif1α* resulted in an increase in E-cadherin mRNA expression in the mice that received either the sulindac or control feed. Also, E-cadherin protein expression was increased in *Hif1α^ΔIEC^* mice, but only in the mice receiving the control feed. In sulindac-treated *Hif1α^ΔIEC^* mice, this increase was abolished.

We have previously shown that sulindac sulfide can induce pro-inflammatory NF-κB and AP-1 signalling, and concurrent apoptosis in the same experimental conditions in colon cancer cells (Mladenova et al., 2011, 2013). Here, we show that the upregulation of EMT-promoting factors c-Jun and IL8 through sulindac sulfide is likely to be the result of activation of AHR, the major pathway that is activated by environmental carcinogens and pharmacological drugs. Sulindac is a known ligand and agonist of AHR (Ciolino et al., 2006; Safe et al., 2013), and the prototype AHR-target gene *CYP1A1* is also upregulated by sulindac sulfide. In MCF7 breast epithelial cells, AHR activation through dioxin leads to activation of Jun NH_2_-terminal kinase (JNK), a reduction in E-cadherin protein levels and the loosening of cell-cell contacts (Diry et al., 2006). Sulindac sulfide is also known to cause phosphorylation of JNK (Singh et al., 2011), which we have confirmed in HCT15 colon cancer cells (data not shown). Thus, sulindac sulfide might reduce E-cadherin protein levels through activation of JNK targets, such as c-Jun, through the AHR pathway.

Sulindac sulfide is known to cause degradation of β-catenin, and this is thought to contribute to its chemopreventive effects (Han et al., 2008; Rice et al., 2003). Here, we show that the reduction in β-catenin protein level in sulindac-sulfide-treated cells is associated with the reduction of E-cadherin and p120-catenin, two proteins controlling cell-cell adhesion. Also, β-catenin-target genes *Lgr5* and *Axin 2* were downregulated in sulindac-induced inflammatory lesions. It remains unexplained as to why the reduction in E-cadherin appears to be more pronounced in the absence of HIF1α signalling in colon epithelial cells. It is possible that this is due to some functional interaction between the AHR and HIF1α pathways (Nie et al., 2001), which share the same binding partner HIF1β (ARNT). Dioxin can also activate AP-1 transcription factors and reduce E-cadherin protein levels independently of HIF1β through non-canonical AHR pathways (Dietrich and Kaina, 2010). Here, we show that both AHR and HIF1α are transcriptionally activated through sulindac sulfide and that this upregulation is abolished by AHR knockdown.

In summary, this study shows that HIF has both pro-inflammatory and protective roles in the proximal colon of sulindac-treated mice. We found that loss of *Hif1α* expression in the *Hif1α^ΔIEC^* mice protects the colon mucosa against sulindac-induced tissue damage and inflammation but that sulindac treatment also causes a defect of E-cadherin protein expression in the proximal colon of these mice. Thus, this study further clarifies the molecular mechanism of sulindac-induced tissue toxicity in the mouse proximal colon.

## MATERIALS AND METHODS

### Generation of *Hif1α^ΔIEC^* mice and administration of sulindac

*Hif1α^F/F^* mice (Ryan et al., 2000) were first crossed with *Villin-Cre* (*VIL*-*Cre*) mice [B6.SJLTg(vil-cre)997Gum/J (Madison et al., 2002); Jackson Laboratories, Bar Harbor, Maine]. Heterozygous *VIL-Cre-Hif1α^F/+^* mice were then backcrossed with *Hif1α^F/F^* to obtain *VIL-Cre-Hif1α^F/F^*, wild-type (*WT*)*-Hif1α^F/F^* and the corresponding *Hif1α^F/+^* heterozygotes. The Mendelian ratios of the four genotypes were 0.22 (*VIL-Cre-Hif1α^F/F^*), 0.32 (*WT-Hif1α^F/F^*), 0.20 (*VIL-Cre-Hif1α^F/+^*) and 0.26 (*WT-Hif1α^F/+^*) of 269 mice born. *VIL-Cre-Hif1α^F/F^* mice are deficient for *Hif1α* in the intestinal epithelial cells (ΔIEC), and the sibling controls *WT-Hif1α^F/F^* retain HIF1α expression from two floxed alleles. The intestinal epithelium *Hif1α*-deficient mice were designated *Hif1α^ΔIEC^* and the sibling control mice *Hif1α^F/F^*. Mice were bred at specific-pathogen-free conditions. *Hif1α^ΔIEC^* mice appeared normal and healthy with similar weight gain compared to *Hif1α^F/F^* mice. Cre-mediated recombination for the HIF1α conditional mutant was determined by using q-PCR analysis of colon mucosal DNA as previously described (Karhausen et al., 2004; Mladenova et al., 2011). The strain background of the *Villin-Cre* mice was C57BL/6J, and the parent *Hif1α^F/F^* mice were at least 91% C57BL/6J and the rest 129S1/SvImJ or 129X1/SvJ (JAX Mouse Diversity Genotyping Array).

Six-week-old mice were treated for 20 weeks with 320 parts per million (ppm) sulindac in the diet before colon collection, as previously described (Mladenova et al., 2011). Mice that were given control food were age-matched with mice on the sulindac diet. Both males and females were used. After examination under a dissecting microscope, individual biopsies from standardised areas of the mouse colon were collected. From each colon, six standard biopsies of flat mucosa were collected, and every visible lesion was dissected. The ‘Australian code for the care and use of animals for scientific purposes' was followed in all experimentation and the project was approved by the Garvan Institute and St Vincent's Hospital Animal Ethics Committee.

Once preserved in 70% ethanol, mouse colons were opened and flattened longitudinally, and were examined using a Leica stereomicroscope (MZ8, Leica Microsystems GmbH, Wetzlar, Germany) with a fitted grid eyepiece. Visible lesions were carefully measured with a fitted eyepiece grid in two dimensions, and the size was calculated as the surface area (mm^2^). The exact location of lesions along the colon length in reference to the caecum was also recorded. Caecum, proximal P1, P2, middle and distal colon segments were collected precisely from standardised colon regions; caecum (the tip of caecum), P1 (1 cm from the caecum-colon junction), P2 (the end of the V-shaped mucosal folds), middle colon (4 cm from the caecum-colon junction) and distal colon (1 cm from the anus). All visible lesions were also biopsied.

For a subset of mice, the mucosal surface of the proximal colonic tissue was lightly scraped or whole colon biopsies were collected. Tissue was snap-frozen in liquid nitrogen for RNA extraction.

### Histopathology analysis

Histopathology assessment was conducted by an anatomical pathologist (J.E.D.) from de-identified slides, as previously described (Mladenova et al., 2011). The features assessed included: acute and/or chronic inflammation, lymphoid aggregates, hyperplastic and/or degenerative changes of the surface epithelium, crypt architectural distortion, fibrosis and neoplasia – classified as epithelial dysplasia or adenocarcinoma. Inflammation was assessed using a scoring system modified from the literature (Cummins et al., 2008; Vowinkel et al., 2004). Three independent parameters were measured: severity of inflammation, depth of injury/inflammation and crypt damage, and scored as shown below. The total histological score was calculated through summing of the three independent scores with a maximum score of 12. For severity of inflammation, representative tissue images are shown in supplementary material Fig. S1 (0=no inflammation; 1=slight – presence of mucosal inflammatory cell infiltrate without significant distortion of the crypt architecture; 2=erosion – superficial ulceration that involved only the surface epithelium and lamina propria; 3=ulceration – defined as loss of the colonic mucosa associated with an acute inflammatory reaction extending at least through the muscularis mucosae). The depth of inflammation was scored as 0=none; 1=mucosal; 2=mucosal and submucosal; 3=transmural. For crypt damage, representative tissue images are shown in supplementary material Fig. S2 (0=none; 1=only surface epithelium damaged; 2=surface crypt and epithelium damaged; 3=basal 1/3 crypt damaged; 4=basal 2/3 crypt damaged; 5=entire crypt lost and surface epithelium damaged; 6=entire crypt and epithelium lost; grades 3 and 4 are not a feature of this model).

### Immunohistochemistry analysis

Sections from tissue paraffin blocks were cut on a Leica microtome. Antigen retrieval was performed in a pressure cooker for 25 s using target retrieval solution at pH 6 (S1699, DAKO, Carpinteria, CA). A Dako autostainer was used for immunohistochemistry. The protocol included an initial step of 3% hydrogen peroxide block (K4011, DAKO) for 5 min in order to quench endogenous peroxidase activity, followed by an avidin-biotin block (Biotin Blocking system, X0590, DAKO) and a serum-free protein block for 30 min (X0909, DAKO). A primary mouse antibody against E-cadherin (1:500; BD Transduction Laboratories™, NJ) was biotinylated with Dako ARKTM (Animal Research Kit K3954) following the manufacturer's instructions, and tissues were incubated for 90 min, followed by incubation with peroxidase-labelled streptavidin (LSAB+System-HRP, K0690, DAKO). A detection system using the liquid DAB+ substrate chromogen system as substrate (K3468, DAKO) was subsequently used, followed by counterstaining with Mayer's hematoxylin. Stained tissue sections were coverslipped using Ultramount No. 4 (Fronine Laboratory Supplies, New South Wales, Australia).

The HIF1α expression intensity (H-score) was calculated by summing the products of the percentage of positively stained surface epithelial cells (0-100) and the staining intensity (1, 2 or 3). The slides were scored in a blind analysis by two researchers (D.M. and F.B.).

### Western blot analysis

HCT15 cells (CCL-225; American Type Culture Collection) were treated with 50 µM sulindac sulfide (Sigma-Aldrich) for 1-24 h and analysed by western blotting following our previously established protocols (Mladenova et al., 2011, 2013). Only adherent cells were analysed for protein expression. The membranes were incubated with primary antibodies for 1 h at room temperature or overnight at 4°C (β-actin, 1:10,000, clone AC15, Sigma-Aldrich), phosphorylated c-Jun (Ser73) no. 9164, c-Jun no. 9165 (Cell Signaling Technology), E-cadherin (1:2000, no. 610181, BD Biosciences), β-catenin (1:2000, no. 610153, BD Biosciences), p120-catenin (1:1000, clone H-90, no. sc-13957, Santa Cruz Biotechnology). ImageJ densitometry software (National Institutes of Health) was used for quantitative densitometry analysis.

### AHR knockdown

Knockdown of AHR was performed using three unique 27mer AHR small interfering (si)RNA duplexes as follows: A – 5ʹ-GGAAUGUACAUGAAGCAAUUAGUCT-3ʹ, B – 5ʹ-GGACUAGAAGAUUAGAAACUACCAA-3ʹ, C – 5ʹ-UGCUCGCAACAAAGAAUUAAUUGGT-3ʹ. Non-targeting siRNA (SR30004; OriGene) was used as control. Transfection was performed in triplicate with 10 nM siRNA using X-tremeGENE HP DNA Transfection reagent (Roche Diagnostics) according to the manufacturer's instructions. Cells were treated with 50 µM sulindac sulfide following 24-h transfection and then harvested for mRNA quantification at the indicated time points.

### Q-PCR analysis

RNA was extracted using Qiagen RNeasy mini (Qiagen GmbH, Germany). Q-PCR reactions were performed using SYBRgreen (Applied Biosystems), Taqman (Applied Biosystems) or Universal Probe Library (UPL) assays (Roche Applied Science) on an ABI Prism 7900-HT Real Time PCR system (Applied Biosystems).

### Statistical analysis

Individual lesion sizes and the total inflammation scores between groups were compared using Student's *t*-test with Welch's correction. The extent of crypt damage between groups was compared using Mann–Whitney test. For q-PCR analyses, the data are graphed as mean±s.e.m. from at least three independent experiments. Student's *t*-test was used for comparisons between two groups. A *P*-value of <0.05 was considered significant.

## Supplementary Material

Supplementary Material
