# Supplementary material for: HIF1α deficiency reduces inflammation in a mouse model of proximal colon cancer
Source: Dis Model Mech. 2015 Sep 1;8(9):1093–103. doi: 10.1242/dmm.019000 (PMC4582097; doi:10.1242/dmm.019000)
Supplement: Supplementary Material [file supp_8_9_1093__index.html]

Supplementary Material 

# HIF1α deficiency reduces colon inflammation in a mouse model of proximal colon cancer

## DMM019000 Supplementary Material

- Supplementary Material
