# Supplementary material for: HIF1α deficiency reduces inflammation in a mouse model of proximal colon cancer
Source: Dis Model Mech. 2015 Sep 1;8(9):1093–103. doi: 10.1242/dmm.019000 (PMC4582097; doi:10.1242/dmm.019000)
Supplement: Supplementary Material [file supp_019000_DMM019000supp.pdf]

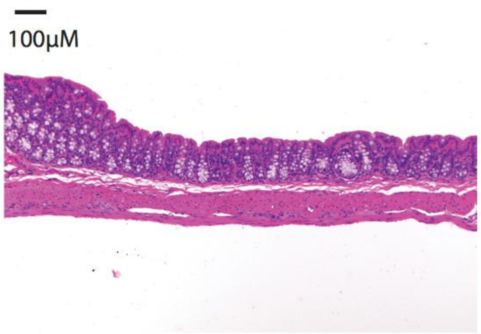

A0C0 = normal

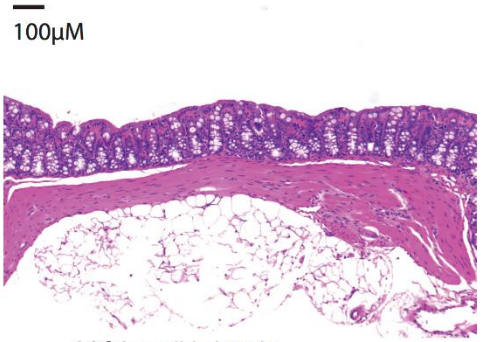

A0C1 = mild chronic
mucosal inflammation

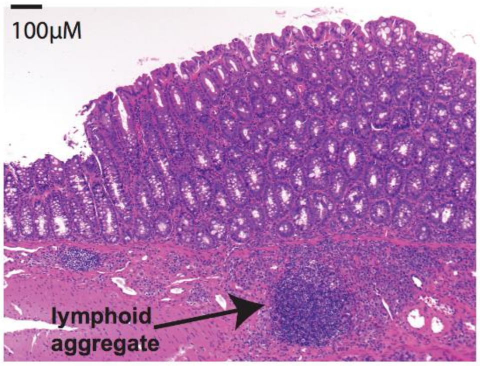

A1C2 = mild acute, moderate chronic
inflammation mucosa and submucosa

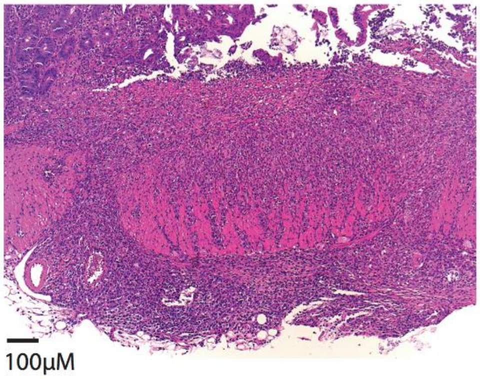

A3C3 = severe acute, severe chronic
inflammation = full thickness

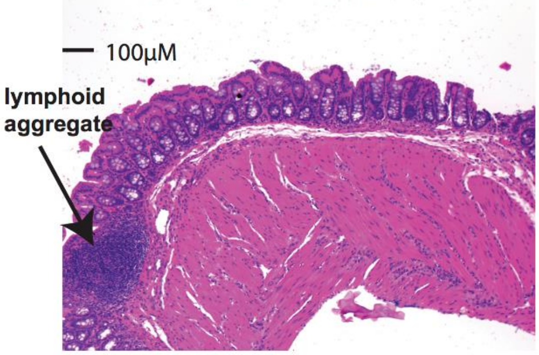

A1C1 = mild active chronic mucosal inflammation
with extension of chronic inflammatory cells
into muscularis propria

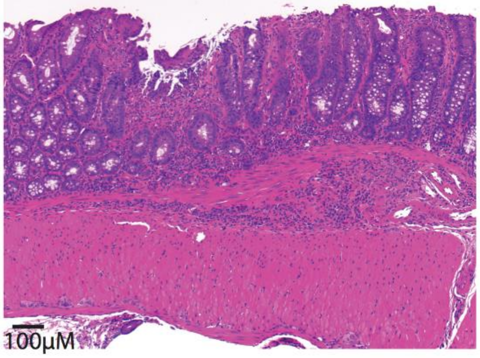

A2C2 = moderate active
chronic inflammation - surface erosion

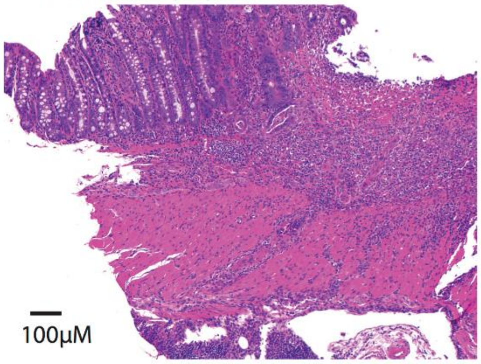

A3C2F0 = severe active chronic inflammation - ulceration

Supplementary Figure 1.
Representative images for the scoring of 'severity of inflammation' in the mouse proximal colon.

Hif1αΔIEC and Hif1αF/F mice were given 320 ppm sulindac in the diet for 20 weeks, after which biopsies were taken from the flat mucosa and lesions along the entire length of the colon and assessed by a Pathologist as described in Methods. A=acute inflammation; C=chronic inflammation.

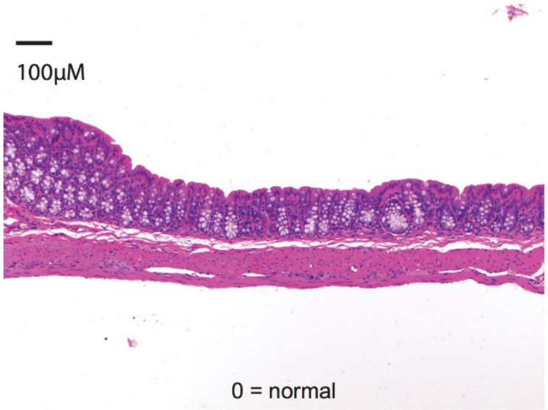

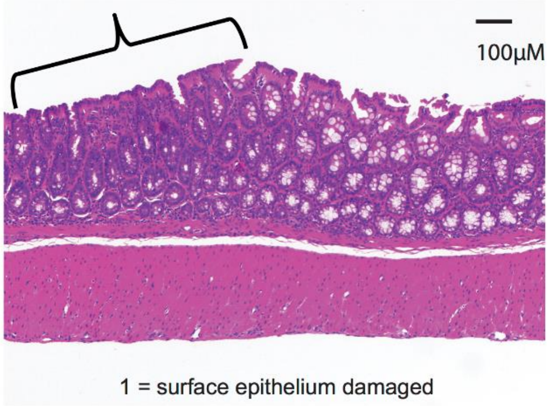

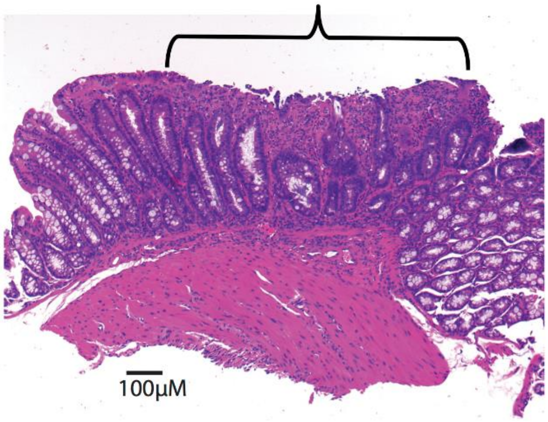

2 = surface crypt and epithelium damaged

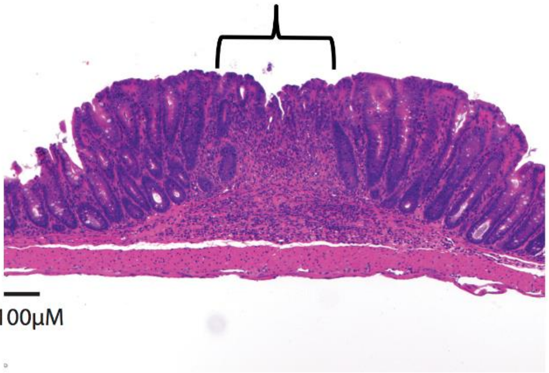

5 = entire crypt lost and surface epithelium damaged

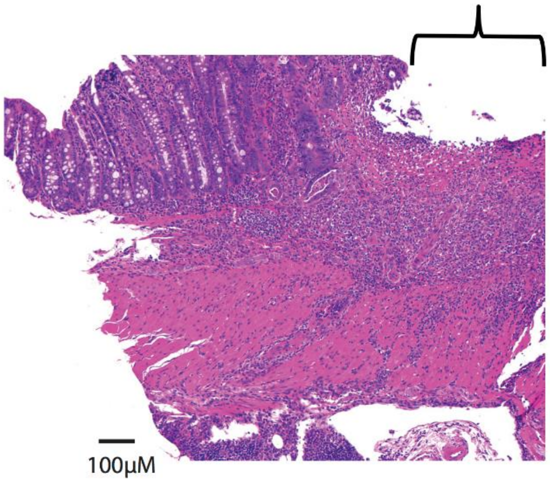

6 = entire crypt and epithelium lost

Supplementary Figure 2.
Representative tissue images for the scoring of 'crypt damage' in the mouse proximal colon.

Hif1αΔIEC and Hif1αF/F mice were given 320 ppm sulindac in the diet for 20 weeks, after which biopsies were taken from the flat mucosa and lesions along the entire length of the colon and assessed by a Pathologist as described in Methods.
